# Metabolic Reprogramming of Host Cells in Response to Enteroviral Infection

**DOI:** 10.3390/cells9020473

**Published:** 2020-02-18

**Authors:** Mei-Ling Cheng, Kun-Yi Chien, Chien-Hsueh Lai, Guan-Jie Li, Jui-Fen Lin, Hung-Yao Ho

**Affiliations:** 1 Department of Biomedical Sciences, College of Medicine, Chang Gung University, Taoyuan 33302, Taiwan; chengm@gap.cgu.edu.tw; 2 Healthy Aging Research Center, Chang Gung University, Taoyuan 33302, Taiwan; 3 Metabolomics Core Laboratory, Healthy Aging Research Center, Chang Gung University, Taoyuan 33302, Taiwan; rflin@mail.cgu.edu.tw; 4 Graduate Institute of Biomedical Sciences, College of Medicine, Chang Gung University, Taoyuan 33302, Taiwanbloodlabor1845@gmail.com (G.-J.L.); 5 Clinical Metabolomics Core Laboratory, Chang Gung Memorial Hospital at Linkou, Taoyuan 33305, Taiwan; 6 Department of Biochemistry and Molecular Biology, College of Medicine, Chang Gung University, Taoyuan 33302, Taiwan; 7 Department of Medical Biotechnology and Laboratory Science, College of Medicine, Chang Gung University, Taoyuan 33302, Taiwan; hanfane@yahoo.com.tw; 8 Research Center for Emerging Viral Infections, Chang Gung University, Taoyuan 33302, Taiwan

**Keywords:** metabolomics, enterovirus, CAD

## Abstract

Enterovirus 71 (EV71) infection is an endemic disease in Southeast Asia and China. We have previously shown that EV71 virus causes functional changes in mitochondria. It is speculative whether EV71 virus alters the host cell metabolism to its own benefit. Using a metabolomics approach, we demonstrate that EV71-infected Vero cells had significant changes in metabolism. Glutathione and its related metabolites, and several amino acids, such as glutamate and aspartate, changed significantly with the infectious dose of virus. Other pathways, including glycolysis and tricarboxylic acid cycle, were also altered. A change in glutamine/glutamate metabolism is critical to the viral infection. The presence of glutamine in culture medium was associated with an increase in viral replication. Dimethyl α-ketoglutarate treatment partially mimicked the effect of glutamine supplementation. In addition, the immunoblot analysis revealed that the expression of glutamate dehydrogenase (GDH) and trifunctional carbamoyl-phosphate synthetase 2, aspartate transcarbamylase, and dihydroorotase (CAD) increased during infection. Knockdown of expression of glutaminase (GLS), GDH and CAD drastically reduced the cytopathic effect (CPE) and viral replication. Furthermore, we found that CAD bound VP1 to promote the de novo pyrimidine synthesis. Our findings suggest that virus may induce metabolic reprogramming of host cells to promote its replication through interactions between viral and host cell proteins.

## 1. Introduction

Enteroviruses are a group of RNA viruses belonging to the family *Picornaviridae* [1]. The virion consists of an icosahedral capsid enclosing a single positive-strand genomic RNA molecule. The capsid is made up of 60 protomers, each of which is constituted of structural proteins VP1, VP2, VP3, and VP4. Enterovirus 71 (EV71) and coxsackievirus A16 are important infectious agents of hand-foot-and-mouth disease (HFMD), and are transmitted through feces, respiratory droplets and saliva of patients [2]. The HFMD is usually self-limiting and is characterized by a mild fever and the presence of oral cavity ulcers, herpangina and papulovesicular rash on extremities. Few patients develop such neurological complications as aseptic meningitis, encephalitis, and acute flaccid paralysis, and cardiopulmonary manifestations. EV71 infection has been endemic in Asia–Pacific regions, including China, Taiwan, Hong Kong, Malaysia, Singapore, Japan, and Korea [2,3,4]. The largest outbreak has occurred in China, with about 3 million cases and 1500 deaths being reported [5,6].

Host metabolic activity is essential to propagation of virus. It is no wonder that virus induces reprograming of host cell metabolism to support viral replication. Human cytomegalovirus virus (HCMV)-infected cells display an increased dependence on glucose, and upregulate their glycolysis and lactate production [7,8]. Glucose depletion is inhibitory to viral replication during HCMV infection. Lipogenesis also increases in the infected cells [9]. Influenza virus-infected host cells have significant alterations in glycolysis, fatty acid biosynthesis, cholesterol metabolism, and nucleotide metabolism [10,11,12]. Likewise, changes in host cell metabolism are associated with adenovirus [13], dengue virus [14], chikungunya virus [15], Zika virus [16], hepatitis B virus [17,18,19], and hepatitis C virus [20,21]. It has been recently found that echovirus 30—another enterovirus—induces changes in host cell metabolism [22]. Little is known about metabolic reprogramming in EV71-infected cells. Our previous study has shown that the mitochondrial functions and redox homeostasis are significantly altered in EV71-infected cells [23]. The central role of mitochondria in metabolism implies that EV71 may induce changes in host cell metabolism. 

It is not completely understood how viruses induce metabolic reprogramming in host cells. Different viruses may adopt different strategies in doing so. Expression of carbohydrate response element binding protein (ChREBP) is upregulated in HCMV-infected cells, which induces glucose transporter type 4 (GLUT-4) expression [24,25]. Activation of AMP-activated protein kinase (AMPK) in the infected cells promotes glycolysis [26]. Cleavage and activation of sterol regulatory element binding protein (SREBP) 1 and 2 also occur in these cells to enhance the expression of lipogenic enzymes [27,28]. Dengue virus non-structural protein 3 (NS3) is able to re-localize the fatty acid synthase (FAS) to the viral replication site, and activates its activity [29]. 

In the present study, we studied the global metabolic changes in EV71-infected Vero cells. Metabolite profiling shows that a number of metabolic pathways, including glutathione metabolism, glycolysis and tricarboxylic acid cycle, change significantly upon EV71 infection. Glutamine/glutamate metabolism plays important roles in EV71 infection. The presence of glutamine in culture medium promotes EV71 replication. Glutamine metabolism-related enzymes, such as GDH and the carbamoyl-phosphate synthetase 2, aspartate transcarbamylase, and dihydroorotase (CAD), increase in expression with time after infection. RNA silencing of *GLS*, *GDH* and *CAD* genes suppresses CPE and EV71 replication. Immunoprecipitation and proteomics analysis revealed an interaction between CAD and the viral protein VP1. Exogenous VP1 expression or EV71 infection increases the flux of CAD reaction. Pharmacological inhibition of pyrimidine biosynthesis suppresses EV71 replication. These findings suggest that EV71 induces metabolic reprogramming in host cells to its own advantage. Interaction between VP1 and CAD may account for an increase in CAD activity.

## 2. Materials and Methods

### 2.1. Cell Culture and Cell Viability Determination 

Vero cells (ATCC CCL-81) were cultured as previously described [30]. In brief, they were maintained in modified Eagle’s medium (MEM) containing 10% fetal calf serum, 100 U/mL penicillin, 0.1 mg/mL streptomycin, and 0.25 μg/mL amphotericin at 37 °C in humidified atmosphere of 5% CO_2_. Cell viability was determined using the neutral red assay, as previously described [31].

### 2.2. Virological Techniques

EV71 prototypic strain BrCr (ATCC VR784) was cultivated in Vero cells, as previously described [30]. Briefly stated, Vero cells were cultured in T25 flask. After reaching a confluency of 80%, the culture was washed twice with phosphate buffered saline (PBS), and inoculated with virus at 37 °C for 1 h. The medium was replaced with the fresh one supplemented with 1% FCS and antibiotics. When over 90% of cells showed CPE, the virus-containing medium was collected, and cell debris was removed by centrifugation. Any remaining viral particles were retrieved from the cell debris by successive freeze-thaw cycles and centrifugation. The plaque reduction assay was performed, as previously described [32,33]. In brief, about 3.5 × 10^5^ cells were seeded per well in a six-well culture plate, and after an overnight incubation, the cells were infected with 100 plaque forming units (PFU) of virus in the medium containing the indicated concentrations of glutamine or BRQ. After 1 h, the cells were washed with PBS, and overlaid with 0.3% agarose in MEM/2% FCS, which was supplemented with the indicated concentrations of glutamine or BRQ. Ninety-six hours later, the cells were fixed with 10% formaldehyde, and stained with 1% crystal violet solution. The plaque number was determined. For quantification of EV71 genomic RNA, quantitative reverse-transcription polymerase chain reaction (qRT-PCR) was performed, as previously described [34]. Total RNA was isolated from the EV71-infected cells using Viral RNA Extraction Miniprep kit (Viogene, Taipei) according to the manufacturer’s instruction. One μg of total RNA was converted to cDNA using RevertAid first-strand cDNA synthesis kit (Thermo Fisher Scientific, Waltham, MA, USA). One μL of a 1:100 dilution of cDNA sample was mixed with LightCycler FastStart DNA Master SYBR Green I (Roche Life Science, Indianapolis, IN, USA) containing the forward and reverse primers for EV71 genome and β-actin (as a control). The forward and reverse EV71 primers were 5’-ACTGACCAAGGACACTTCAC-3’ and 5’-CCAGTGTGAGTTCCAAGTTT-3’, respectively. The forward and reverse β-actin primers were 5’-ATCGTGCGTGACATTAAGGAG-3’ and 5’-CCATCTCTTGCTCGAAGTCC-3’, respectively. The PCR reaction was performed with a LightCycler 1.5 instrument (Roche Life Science, Indianapolis, IN, USA). The CPE was determined microscopically, as previously described [30]. Cells were stained with Hoechst 33342 dye at a concentration of 5 μg/mL for 15 min and examined under fluorescence microscope. The percentage of cells with CPE (i.e., chromatin condensation and formation of crescent-shaped nuclei) was quantified using IN Cell Analyzer 1000 (GE Heathcare Life Sciences, Chicago, USA), as previously described [32,33].

### 2.3. RNAi Knockdown 

The siRNA transfection was performed using Lipofectamine 2000 according to the manufacturer’s instruction. Briefly stated, around 3.5 × 10^5^ cells were seeded in a well of six-well culture plate. Twenty-four hours later, 2.5 μL of Lipofectamine 2000 (Thermo Fisher Scientific, Waltham, MA, USA) was incubated with 50 pmol of ON-TARGETplus SMARTpool siRNA (Dharmacon, Lafayette, CO, USA) against GDH (L-004032-00-0010), GLS (L-004548-01-0010) or CAD (L-009471-00-0010), or with 50 pmol of ON-TARGETplus non-targeting pool siRNA (D-001810-10-50), in Opti-MEM I Reduced Serum Medium (Thermo Fisher Scientific, Waltham, MA, USA) for 20 min at room temperature. The siRNA-Lipofectamine 2000 complex was added to cells. The transfected cells were further incubated at 37 °C for 48 h. 

### 2.4. Plasmid DNA and DNA Transfection

The vector encoding the Myc-tagged fusion of GFP and VP1 (GFP-VP1-Myc) was constructed by insertion of a PCR-amplified VP1 fragment (derived from Tainan/4643/TW strain) in between BspEI and XbaI sites of the vector pcDNA3.1(+)/myc-His-GFP. The latter itself was generated by cloning of a PCR-amplified EGFP fragment in between HindIII and XbaI sites of the vector pcDNA3.1(+)/myc-His B. The vector encoding N-FLAG-tagged CAD (N-FLAG-CAD) was constructed by cloning of the N-FLAG-CAD coding sequence from pQC-XIP.n3×Flag.CAD in between NotI and NruI sites of the vector pcDNA3.1(+)/myc-His B. The pQC-XIP.n3×Flag.CAD vector was constructed by insertion of a PCR-amplified CAD in between PsiI and MluI sites of pQC-XIP.3×Flag vector. The CAD coding sequence was amplified from the clone pCS6(BC065510)-seq (TransOMIC technologies, Huntsville, AL, USA). Similarly, the vector encoding C-FLAG-tagged CAD (C-FLAG-CAD) was constructed by cloning of the C-FLAG-CAD coding sequence from pQC-XIP.CAD.c3×Flag in between NotI and PmeI sites of pcDNA3.1(+)/myc-His B. The pQC-XIP.CAD.c3×Flag vector was generated by insertion of the PCR-amplified CAD in between NotI and NaeI sites of pQC-XIP.3×Flag vector. The control vector pC34V has been previously described [35]. This vector expresses a fusion protein (C34V) of Cerulean protein and Venus protein, which are joined by a linker containing a tobacco etch virus NIa protease (TEVp) recognition sequence and a FLAG epitope. 

DNA transfection was performed with JetPRIME in accordance to the manufacturer’s instruction. In brief, 3.5 × 10^5^ cells were seeded in a well of six-well culture plate. After 24 h, 2 μg of plasmid DNA was diluted with 200 μL of JetPRIME buffer. The diluted DNA was incubated with 4 μL of JetPRIME reagent at room temperature for 10 min. The DNA complex was added to cells. The transfection medium was replaced with fresh medium 6 h later. The transfected cells were incubated at 37 °C for an additional 18 or 42 h. 

### 2.5. Metabolomic Analysis 

10^6^ Vero cells were seeded in 10 cm culture dish. After 24 h, the medium was replaced with serum-free MEM, and the cells were infected without or with EV71 at the indicated multiplicity of infection (MOI) at 37 °C for 1 h. An equal volume of MEM supplemented with 2% FCS was added. Sixteen hours later, medium was removed, and the cells were washed twice with PBS. The cell monolayer was scraped on ice in the 80% methanol/0.1% formic acid solution which was pre-cooled to −80 °C. The cell lysate was vortexed and centrifuged at 15,300× *g* for 15 min, and the supernatant was retained. The precipitate was extracted once more, and the extracted fractions were combined. The extract was dried under a stream of nitrogen gas and dissolved in 150 μL of 0.1% formic acid. After a further centrifugation (15,300 × *g* for 15 min), 130 μL of supernatant was retained for metabolomic analysis. 

Liquid chromatography-time-of-flight-mass spectrometry (LC-TOF-MS) was performed, as previously described [36]. Liquid chromatographic separation was performed on an ACQUITY 1.8 μm HSST3 C18 column (100 mm × 2.1 mm) (Waters Corp., Milford, MA, USA) using an ACQUITY Ultra Performance Liquid Chromatography (UPLC) system (Waters Corp., Milford, MA, USA). The column temperature and flow rate were set at 40 °C and 0.4 mL/min, respectively. The sample elution was performed using a gradient profile: 0–4.0 min, 1–50% B; 4.0–5.0 min, 50%–98% B; 5.0–7.4 min, 98% B; 7.5–10.0 min, 1% B (for re-equilibration). Solvent A was 0.1% formic acid in water, and solvent B was 0.1% formic acid in acetonitrile. Mass spectrometry was performed on a Waters SYNAPT G2S Q-TOF-MS (Waters MS Technologies, Manchester, UK) operated in positive and negative ion modes. The scan range was from 50–990 *m*/*z*. The desolvation temperature and gas flow rate were set at 500 °C and 1000 L/h, respectively. The source temperature was 150 °C. The capillary voltage was set at 3000 V for positive ion mode or 2000 V for negative ion mode. The cone voltage was 25 V. Leucine encephalin, generating [M-H]^+^ ion (*m*/*z* 120.0813, 556.2771) for positive ion mode or [M-H]^-^ ion (*m*/*z* 236.1035, 554.2615) for negative ion mode, was used as the lock mass at a concentration of 200 ng/mL and a flow rate of 10 μL/min. 

### 2.6. Processing of Metabolomics Data and Metabolite Validation

All spectral data were processed for peak picking, alignment and normalization, using Progenesis QI data analysis software (Nonlinear Dynamics, Newcastle, UK). The peak intensities of all features were obtained. The features were identified through search in Metabolite Link (METLIN) [37] and Human Metabolome (HMDB) [38] databases, and/or by comparison to the retention times and mass spectra of standard compounds. The MS data were subjected to principal component analysis (PCA) and orthogonal partial least-squares discriminate analysis (OPLS-DA) using statistical package SIMCA-P^+^ (Umetrics, Sweden).

For validation of the metabolites, the standard compounds were subjected to chromatographic separation, and MS and MS/MS under the condition identical to that of metabolite profiling. MS/MS spectra were collected at a speed of 0.3 s per scan, with a medium isolation window of 4 *m*/*z*. The trap collision energy was set from 5 to 35 V. 

### 2.7. Immunoprecipitation and Proteomic Analysis

Vero cells were transfected with expression vectors encoding the GFP-VP1-Myc and GFP-Myc (c-Myc-tagged GFP as control), as described above. The culture medium of the transfected cells was removed. After two washes in PBS, the cells were scraped off the culture plate, collected in a microfuge tube, and centrifuged at 16,000 × *g* for 10 min at 4 °C. The cell pellet was lysed in lysis buffer (catalog number 240107; Agilent, Santa Clara, CA, USA) supplemented with cOmplete Mini EDTA-free protease inhibitor cocktail (Roche, Mannheim, Germany), and was subjected to three freeze-thaw cycles. The Dynabead Protein G magnetic beads (Thermo Fisher Scientific, Waltham, MA, USA) were processed according to the manufacturer’s instruction, and were bound to 2 μg of c-Myc monoclonal antibody (9E10) (Thermo Fisher Scientific, Waltham, MA, USA) for 10 min at room temperature. After antibody binding, the beads were washed gently with PBS (pH 7.4)/0.02% Tween 20. The magnetic beads were separated with a magnet, and after removal of the wash buffer, the antibody-coated beads were incubated with 500 μg of cell lysate overnight at 4 °C. The protein-bound beads were washed three times with wash buffer and two more times with PBS. The protein was eluted from beads thrice with 50 μL 0.1% trifluoroacetic acid/50% acetonitrile, and the eluates were combined and lyophilized using centrifugal evaporator. The sample was dissolved in 50 mM NaHCO_3_ (pH 8.0); treated with 10 mM dithiothreitol at 57 °C for 1 h; and alkylated with 10 mM iodoacetamide at room temperature for 30 min. The sample was then digested with trypsin (sequencing grade; Promega, Madison, WI, USA) at an enzyme/substrate ratio of 1:20 at 37 °C overnight. The digested samples from EV71-infected and control cells were labeled with heavy (^13^CD_2_O) and light (^12^CH_2_O) formaldehyde in the presence of sodium cyanoborohydride. The reaction was allowed to proceed for 1 h at 37 °C and quenched by the addition of ammonium bicarbonate (at final concentration of 35 mM). The dimethylated peptides were mixed and dried. The dried sample was dissolved in 0.1% formic acid and desalted using RP micro-column (Source 15RPC; GE Healthcare Life Sciences, Pittsburgh, PA, USA). The sample was subjected to Ultimate 3000 two-dimensional strong cation exchange (SCX)/reverse-phase nanoscale liquid chromatography (LC) coupled with Orbitrap Elite hybrid mass spectrometer (Thermo Electron, Bremen, Germany). The peptides were fractionated on a SCX column (0.38 × 100 mm, Luna SCX, 5 μm; Phenomenex, Torance, CA, USA), using an ammonium chloride gradient with 30% acetonitrile, into six fractions over 540 min. During separation, each fraction was diluted online with 0.1 % formic acid, trapped with a RP trapping column, and then separated on a RP column (0.075 × 180 mm, Hrdro-RP, 2.5 μm; Phenomenex, Torance, CA, USA) using an acetonitrile gradient in 0.1% formic acid over 90 minutes. The mass spectrometer was operated in positive ion mode, and the full scan spectra (*m*/*z* 400–2000) were acquired by the orbitrap analyzer, with the resolution set at 60,000. MS/MS spectra were acquired in a data-dependent manner in the ion trap. From each MS spectrum, the top 15 most intense precursor ions with an intensity greater than 5000 were selected for MS/MS fragmentation. Each precursor was sequenced twice and then excluded for 75 s. The MS data were processed using the Proteome Discoverer software (version 1.3.0.339) for protein identification and quantification. The MS/MS spectra were searched against the Swiss-Prot human database (version 2010_6; 20294 sequences) using the Mascot search engine (version 2.2; Matrix Science Inc., Boston, MA, USA). The parameters for database search were set as below: enzyme specificity, trypsin; maximum missed cleavage, 2; fixed modification, carbamidomethylation of cysteine; variable modifications, acetylation of protein N-terminal amino acids, oxidation of methionine, and glutamine to pyroglutamic acid conversion at peptide N-terminus, dimethylation of light and heavy version labels at lysine and peptide N-terminus; mass tolerance, 10 ppm and 0.6 Da for MS and MS/MS spectra, respectively. The false discovery rate of the search result was estimated by searching against a decoy database, and was set to 0.01 for both peptide and protein levels. Finally, proteins identified with at least two unique peptides were considered as confident identifications, and quantification was done by calculating the median of ratios of the corresponding unique peptides of each protein. Any proteins that were differentially co-immunoprecipitated with GFP-VP1-Myc versus with GFP-Myc represent those cellular proteins interacting with VP1. 

For studying the interaction between VP1 and CAD, we transfected Vero cells with expression vectors encoding GFP-VP1-Myc and N-FLAG-CAD (or C-FLAG-CAD). Immunoprecipitation was performed using Dynabead Protein G magnetic beads coated with 2 μg of c-Myc monoclonal antibody (9E10) or FLAG M2 monoclonal antibody (Sigma-Aldrich, St. Louis, Missouri, USA) as described in the preceding section. 

### 2.8. Western Blotting

For western blotting of cell lysate, the cells were lysed in the same lysis buffer as that used in immunoprecipitation. For the analysis of the immunoprecipitation, the protein-bound beads were heated in Laemmli sample buffer at 95 °C for 5 min. The sample was analyzed by western blotting, as previously described [34]. The antibodies to glutaminase (GLS) (ab93434), glutamate dehydrogenase (GDH) (ab55061) and CAD (ab99312) were available from Abcam (Cambridge, UK). The antibody to actin (clone AC-40, A3853) was purchased from Sigma-Aldrich (St. Louis, Missouri, USA). The horseradish peroxidase (HRP)-conjugated secondary antibodies to rabbit (sc-2004) and mouse (sc-2005) IgG were available from Santa Cruz Biotechnology Inc. (Dallas, Texas, USA). The HRP-conjugated anti-c-Myc (R951-25) and anti-FLAG (MA1-91878) antibodies were available from Thermo Fisher Scientific. Rabbit polyclonal anti-EV71-VP1 antibody (PAB7631-D01P) was purchased from Abnova (Neihu District, Taipei City, Taiwan).

### 2.9. Assessment of Cellular CAD Flux 

About 1 × 10^6^ cells were seeded in a T75 culture flask, and mock- or transfected with the indicated vectors 24 h later. After 20 h, the culture medium was replaced with MEM supplemented with 40 mM NaH^13^CO_3_, 10 mM HEPES and 10% FCS. For control, the medium contained the unlabeled NaHCO_3_. The cells were incubated at 37 °C in an incubator without CO_2_ supply for 2 h, and then treated with 20 μM brequinar sodium (BRQ; Sigma-Aldrich, St. Louis, Missouri, USA), dihydroorotate dehydrogenase (DHODH) inhibitor, for 1 h. The cells were extracted, as described in Section 2.5, and the level of [^13^C]-dihydroorotate (*m*/*z* 158.0283) was measured via a targeted metabolomic approach. The level of [^13^C]-dihydroorotate accumulating in the presence of BRQ was divided by the labeling time, and it indicates the average flux of CAD-catalyzed reactions. For the experiment involving the EV71 infection, about 1.2 × 10^6^ cells were seeded in a T75 culture flask, and infected with virus at the indicated MOI for 1 h. The culture medium was replaced with MEM supplemented with 40 mM NaH^13^CO_3_, 10 mM HEPES and 1% FCS. For control, the medium contained the unlabeled NaHCO_3_. The cells were incubated at 37 °C in an incubator without CO_2_ supply for 2 h, and then treated with 20 μM BRQ for 1 h. The [^13^C]-dihydroorotate was quantified, as described above. 

### 2.10. Statistical Analyses 

The statistical analyses of metabolomic and proteomic data are described in the respective sections. The statistical analyses of other experimental data were performed with GraphPad Prism 5 software (GraphPad Software Inc., San Diego, CA, USA). Two-way analysis of variance (ANOVA) with Sidak’s multiple-comparison test, two-tailed unpaired Student’s t test, a Kruskal-Wallis test with Dunn’s multiple comparison test, and a Mann-Whitney test were used where appropriate. 

## 3. Results

### 3.1. Identification of Metabolic Changes in EV71-Infected Cells

**Figure 1:**
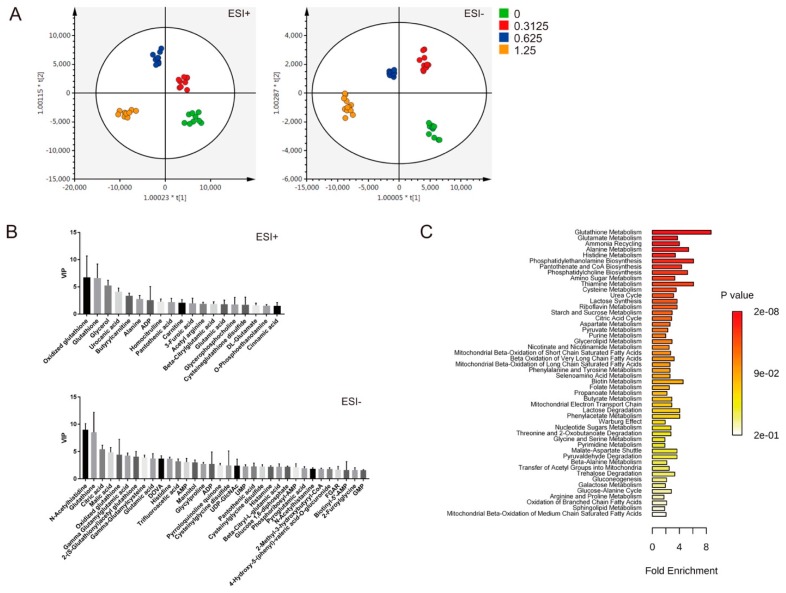
Unique changes in the metabolism of EV71-infected cells. Vero cells were infected without or with EV71 at MOIs of 0.3125, 0.625, or 1.25 for 24 h, and extracted for metabolomic analysis with UPLC-Q-TOF-MS operated in electrospray positive (ESI+) and negative (ESI-) modes (n = 9). The datasets were analyzed by OPLS-DA, and the score plots for the features obtained in ESI+ and ESI– modes are shown (**A**). Some of the identified metabolites with VIP scores >1.5 are shown (**B**). The summary plot for metabolite set enrichment analysis for EV71-infected cells (**C**).

To study the metabolic changes associated with EV71 infection, we infected Vero cells with EV71 at an MOI of 0, 0.3125, 0.625, or 1.25, and extracted their metabolites for metabolomic analysis. The cell extracts were analyzed by UPLC-Q-TOF-MS operated in electrospray ionization (ESI) positive and negative modes. The datasets corresponding to mock-infected cells and those infected at different MOIs were analyzed by partial least squares discriminant analysis (OPLS-DA). The data points corresponding to cells infected at different MOIs were spatially separated from one another and from mock-infected cells, indicating an MOI-dependent change in metabolism of infected cells (Figure 1A). Features that were differentially abundant between these groups were searched against the HMDB and METLIN databases. Those with variable importance in projection (VIP) scores greater than 1.5 were selected for metabolite set enrichment analysis. Examples of such identified features (i.e., metabolites) are shown in Figure 1B. Metabolism of glutathione and amino acids, such as glutamate, aspartate, alanine and histidine, was significantly altered in the infected cells. Other pathways, including tricarboxylic acid cycle and pyruvate metabolism, were also affected (Figure 1C). The metabolites differentially abundant in cells infected at different MOIs are tabulated in Table S1.

### 3.2. Changes in Amino Acid Pools in EV71-Infected Cells 

**Figure 2:**
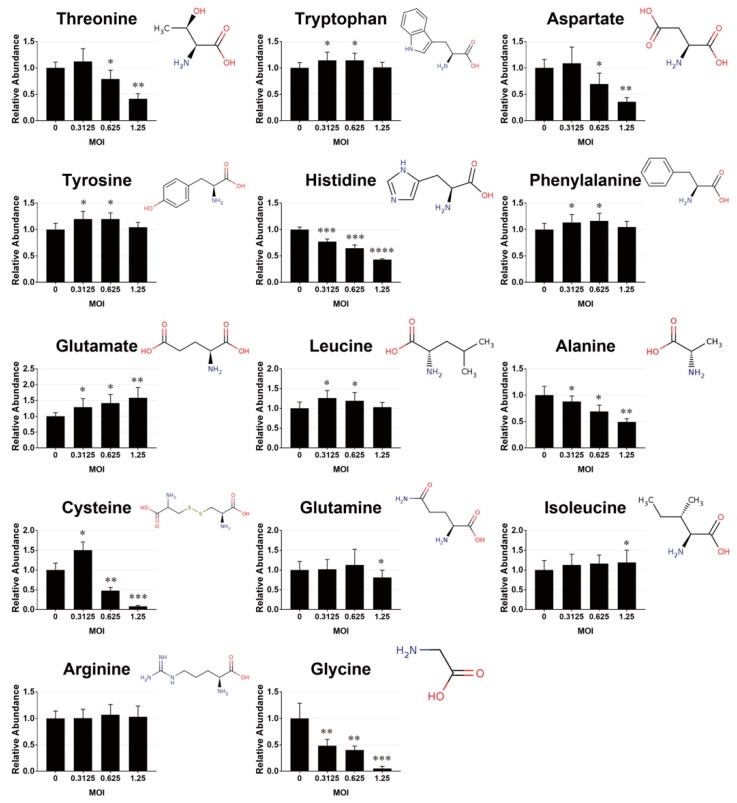
EV71 infection is associated with variations in amino acid pools. Vero cells were infected and extracted for metabolomic analysis, as described in Figure 1. Features corresponding to specific amino acids were extracted. The levels of metabolites are expressed relative to those of mock-infected cells. Data are mean ± SD (n = 9). * *p* < 0.05, ** *p* < 0.01, *** *p* < 0.005, significant difference from mock-infected cells.

A number of amino acids and dipeptides changed in abundance in cells after infection. As shown in Figure 2, the levels of threonine, aspartate, alanine, and glycine decreased, while that of glutamate increased significantly with the MOI of the viral inoculum. The levels of tryptophan, tyrosine, phenylalanine and leucine increased at MOIs of 0.3125 and 0.625 and decreased at MOI of 1.25. Isoleucine level was elevated in cells infected at MOI of 1.25. Cysteine accrued in the cells infected at MOI of 0.3125, and was significantly lowered in abundance at higher MOIs. The level of glutamine that was present in culture medium remained unchanged in infected cells at MOI up to 0.625, and dropped modestly at MOI of 1.25.

### 3.3. Mapping of Metabolites onto Metabolic Pathways 

**Figure 3:**
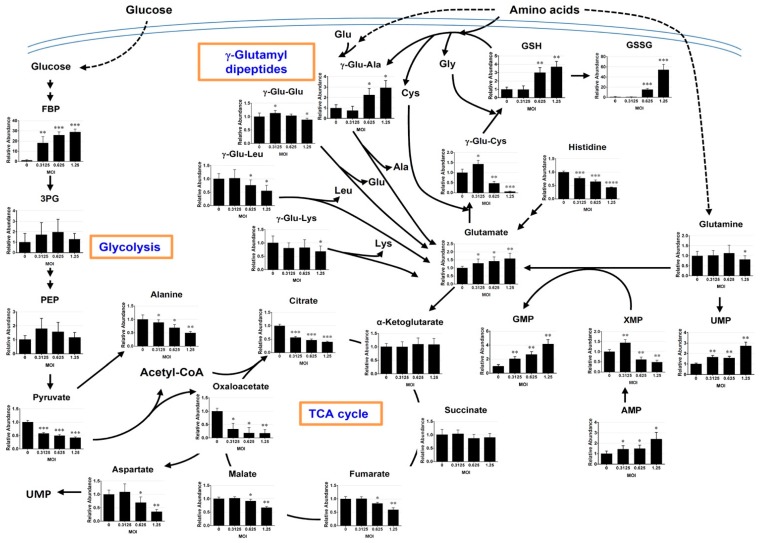
EV71-induced changes in metabolism of epithelial cells. Vero cells were infected and extracted for metabolomic analysis as described in Figure 1. The features of interest were extracted, and the validated metabolites of interest were mapped onto biochemical pathways. The levels of metabolites are expressed relative to those of mock-infected cells. The arrow indicates the metabolic pathway, and the dashed one indicates the transport of biomolecules. Data are mean ± SD (n = 9). * *p* < 0.05, ** *p* < 0.01, *** *p* < 0.005, significant difference from mock-infected cells.

The metabolites of interest were mapped onto metabolic pathways through the use of algorithms available from KEGG and HMDB databases. A number of metabolites related to glutathione, glycolytic intermediates, citric acid cycle intermediates, and purine and pyrimidine nucleosides, changed significantly (Figure 3).

#### 3.3.1. Changes in γ-Glutamyl Dipeptides 

Several γ-glutamyl dipeptides, which are most likely involved in amino acid transport and glutathione metabolism [39], appeared to be affected by infection (Figure 3). The levels of γ-glutamyl cysteine and γ-glutamyl leucine decreased, while that of γ-glutamyl alanine increased with MOI in the infected cells. 

#### 3.3.2. Changes in the Metabolites Related to Glutathione Metabolism

EV71 infection appears to be associated with oxidative stress. The levels of glutathione (GSH) and glutathione disulfide (GSSG) increased with MOI. However, the latter was elevated to a greater extent than the former. The GSSG/GSH ratios of cells infected at MOIs of 0.625 and 1.25 were 5.16 and 14.67, respectively. There were changes in precursors for GSH synthesis in these cells. The levels of cysteine and γ-glutamyl cysteine increased modestly at the MOI of 0.3125 and declined at higher MOIs. Glycine level decreased significantly as MOI increased. These findings suggest that the steady state level of GSH may increase at the expense of its precursors. 

#### 3.3.3. Changes in the Intermediates of Glycolysis and Citric Acid Cycle

The abundance of fructose bisphosphate (FBP), the product of rate-determining step of glycolysis, increased 18-29-fold in the infected cells, depending on MOI. The levels of 3-phosphoglycerate (3PG) and phosphoenolpyruvate (PEP) increased non-significantly at low MOIs. In contrast, the downstream products pyruvate decreased in abundance in the infected cells. These findings suggest that the glycolysis may be enhanced in the infected cells to produce pyruvate, which can be converted to other molecules, such as citrate or oxaloacetate. There were concomitant changes in the intermediates of citric acid cycle. Citrate levels dropped by 45% to 60% in cells infected at MOI ranging from 0.3125 to 1.25. Apparently, the pools of α-ketoglutarate and succinate did not change significantly upon infection. In contrast, the amount of fumarate, malate, and oxaloacetate declined in the infected cells, with the extent of reduction in the former two metabolites being lower than that of oxaloacetate. The levels of fumarate and malate were respectively 17.2% and 8.7% lower in cells infected at an MOI of 0.625, as compared to the mock-infected cells. The oxaloacetate level was lowered by 81.6% in these cells, as compared to that of control. These findings imply that glutamate may feed into citric acid cycle at the point of α-ketoglutarate to maintain the pools of α-ketoglutarate and succinate, and that their downstream products fumarate, malate and oxaloacetate are most likely consumed through reactions other than citrate formation.

#### 3.3.4. Changes in the Purine and Pyrimidine Metabolism

The levels of uridine monophosphate (UMP), adenosine monophosphate (AMP) and guanosine monophosphate (GMP) increased in infected cells. The levels of glycine and aspartate, which are required for purine and pyrimidine biosynthesis, decreased substantially in the infected cells. Another precursor glutamine, which was supplied in culture medium, did not change significantly in the infected cells, with the exception that it was slightly reduced at the highest MOI used. However, glutamate, the by-product of the glutamine-dependent reactions in purine and pyrimidine biosynthesis, increased significantly in abundance in the infected cells. These findings suggest that the purine and pyrimidine biosynthesis may be up-regulated in the infected cells.

### 3.4. Glutamine is Essential to Viral Replication

**Figure 4:**
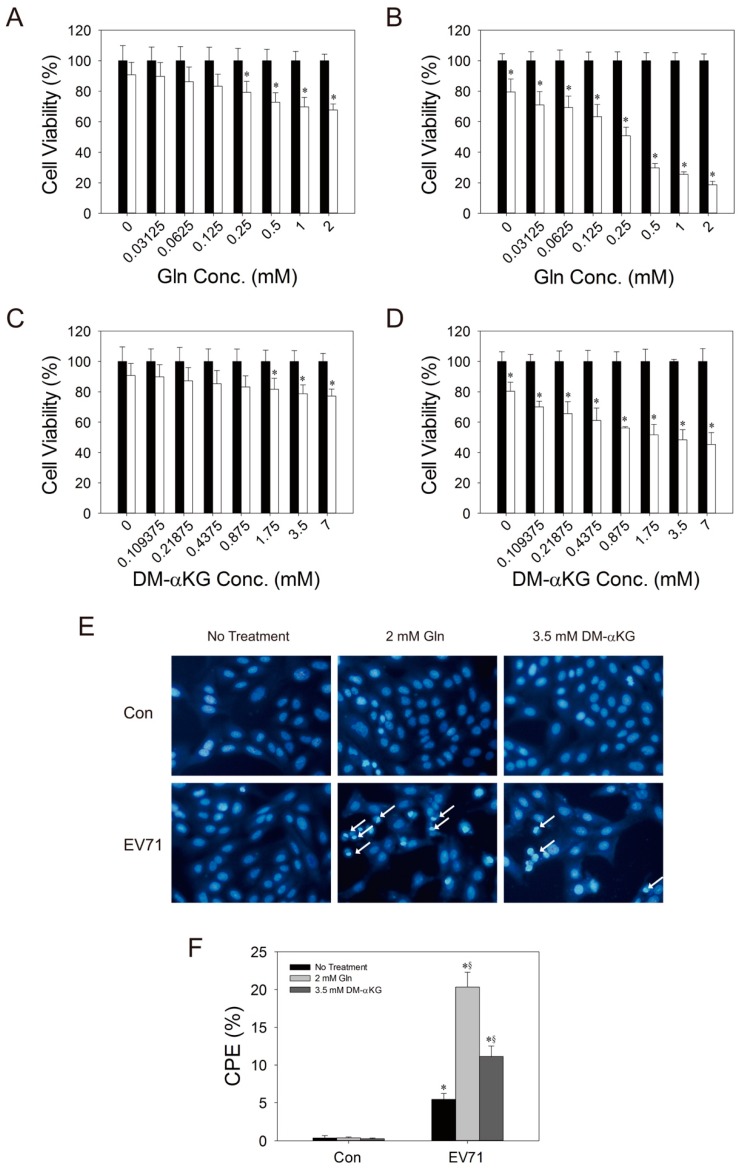
Glutamine deprivation rescues cells from viability loss and cytopathic effect. Vero cells were cultured in medium containing the indicated concentrations of glutamine (Gln) (**A**,**B**), or in glutamine-free medium supplemented with the indicated concentrations of DM-αKG (**C**,**D**). They were mock- (solid bar) or infected (empty bar) with EV71 at MOI of 1.25 for 24 (**A**,**C**) and 48 h (**B**,**D**), and the viability was assayed by neutral red assay. The data are expressed as percentage relative to those of mock-infected cells and presented as mean ± SD of six separate experiments. *p < 0.05, significant difference from mock-infected cells at a specified concentration. (**E**) Cells were cultured in glutamine-free medium (no treatment), medium containing 2 mM Gln, or glutamine-free medium containing 3.5 mM DM-αKG, and were mock- (Con) or infected (*EV71*) with EV71 for 24 h. They were stained with Hoechst 33342 and examined under a fluorescence microscope. The white arrows indicate cells with CPE. The results shown are representative of six experiments (original magnification: × 200). The cells with CPE are those characterized by chromatin condensation and formation of crescent-shaped nuclei. (**F**) The percentage of such cells, taken as a measure of CPE, was determined by IN Cell Analyzer 1000. Data are means ± SD of three experiments. * *p* < 0.01, significant difference from mock-infected cells for the indicated treatment; ^§^ *p* < 0.05, significant difference from the no-treatment group.

**Figure 5:**
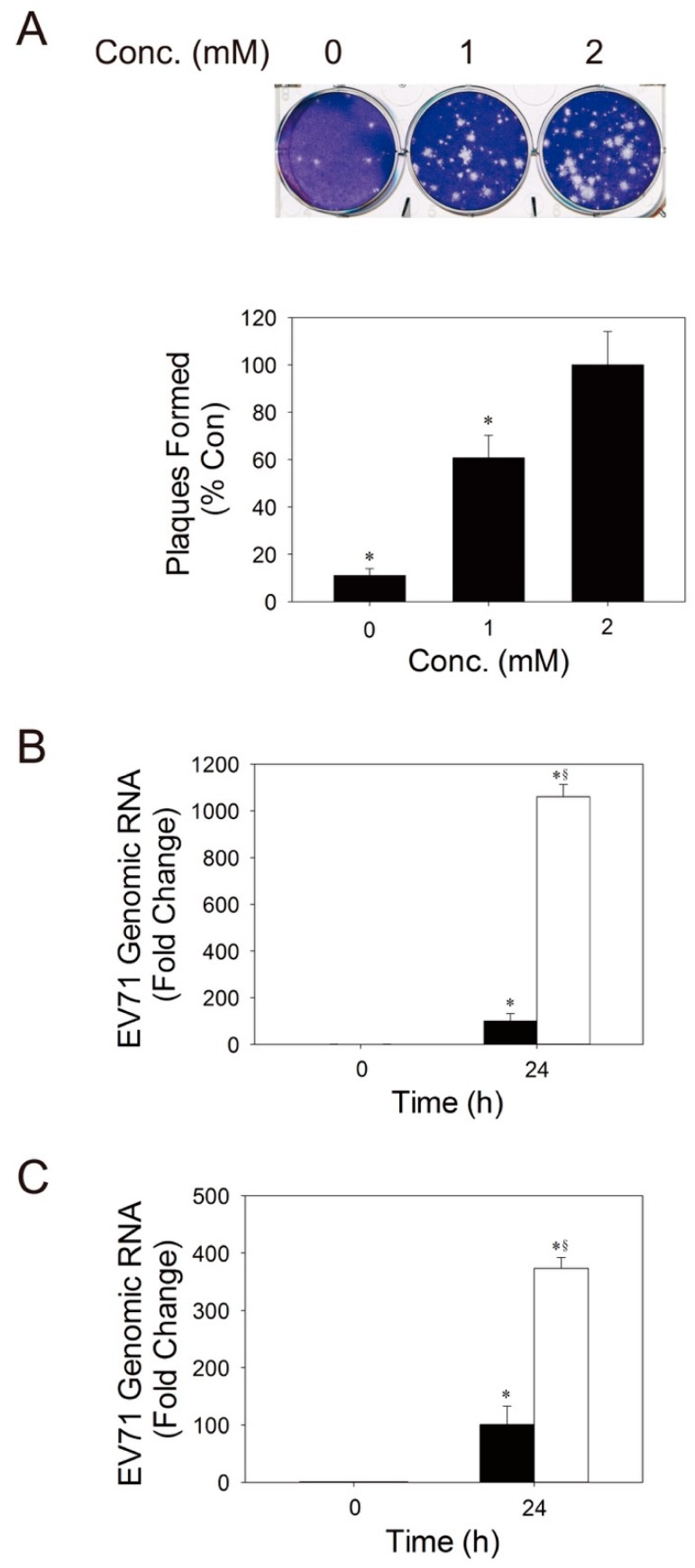
Glutamine and DM-αKG are essential to viral replication. (**A**) Vero cells were infected with EV71 in the medium containing the indicated concentrations of glutamine for 1 h, and were overlaid with 0.3% agarose in MEM/2% FCS, supplemented with the indicated concentrations of glutamine. The infected cells were processed for crystal violet staining as described in Materials and Methods. Representative plates of three experiments are shown here (upper panel). The number of plaques was counted, and is expressed as percentage relative to that formed in the medium supplemented with 2 mM glutamine. Data are mean ± SD. * *p* < 0.01, significant difference from that of 2 mM glutamine group. (**B**,**C**) Vero cells were cultured in medium containing the 0 (solid bar) and 2 (empty bar) mM glutamine (**B**), or in glutamine-free medium supplemented without (solid bar) or with 3.5 (empty bar) mM DM-αKG (**C**). They were infected with EV71 at MOI of 1.25. The total RNA was extracted from cells at 0 and 24 h p.i., and qRT-PCR was performed to determine the level of viral genomic RNA. The result is expressed as fold change relative to that at 0 h p.i. Data are mean ± SD of six experiments. * *p* < 0.01, significant difference from that at 0 h. ^§^ *p* < 0.05, significant difference from the no-treatment group.

As stated earlier, the significant increase in glutamate in infected host cells is indicative of continual uptake of glutamine and its utilization. It is hypothesized that glutamine is essential to viral replication. To test this possibility, we varied the concentration of glutamine in culture medium, and examined its effect on the viability of infected cells. Despite an effect of glutamine on the growth of uninfected cells (Figure S1), its presence was associated with a reduction in the relative viability of the infected cells (Figure 4A,B) and with an increase in the extent of CPE (Figure 4E,F). Glutamine enhanced plaque formation and viral replication (Figure 5A,B). We further tested whether glutamine exerts its effect through its deamidation to glutamate and subsequent conversion to α-ketoglutarate, which enters TCA for energy production. We cultured Vero cells in glutamine-free culture medium, which was supplemented with a cell-permeable ester of α-ketoglutarate, dimethyl-α-ketoglutarate (DM-αKG). The cells were infected, and the effect of DM-αKG was examined. Despite a modest effect of DM-αKG on the growth of uninfected cells (Figure S2), DM-αKG promoted a reduction in the relative viability of the infected cells and enhanced CPE formation, but to a lesser extent than glutamine (Figure 4C,D). The level of viral genomic RNA in cells treated with 3.5 mM DM-αKG was 3.7-fold higher than that of untreated cells at 24 h post-infection (p.i.) (Figure 5C). These findings suggest that glutamine may enhance viral replication in host cells, partly via an increase in supply of TCA intermediates.

### 3.5. Expression of GDH and CAD is Upregulated during EV71 Infection

**Figure 6:**
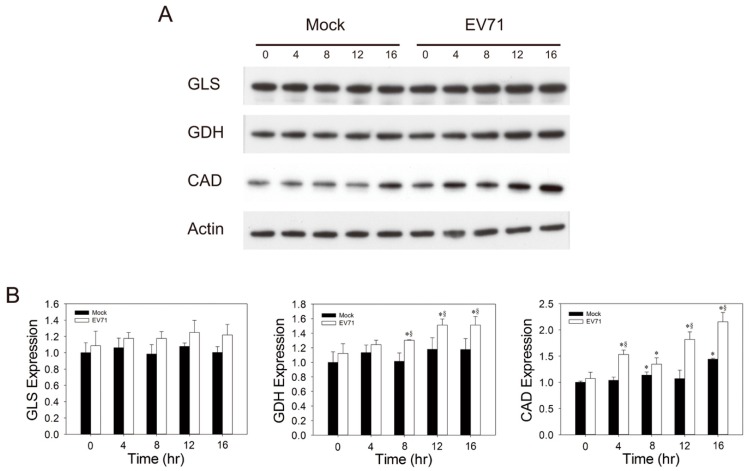
Increased expression of GDH and CAD proteins during viral infection. (**A**) Vero cells were mock- (Mock) or infected with virus (*EV71*) at MOI of 1.25, and were harvested at the indicated time-points p.i. for western blotting with antibodies to GLS, GDH, CAD and actin. A representative result of three experiments is shown. (**B**) The expression levels were quantified by densitometry and Image J software and normalized to that of actin, and are expressed as fold change relative to that of mock-infected cells at 0 h p.i. * *p* < 0.05, significant difference from cells at 0 h; ^§^ *p* < 0.05, significant difference from mock-infected cells at the same time-points.

It is evident that glutamine acts to enhance viral replication through αKG-dependent and independent pathways. To study the exact metabolic pathways involved, we examined the temporal changes in the expression of glutamine metabolism-related enzymes, including glutaminase (GLS), GDH and CAD, in host cells during infection. GLS, in concert with GDH, converts glutamine to αKG; and CAD utilizes glutamine for production of dihydroorotate, a precursor for pyrimidine biosynthesis. As shown in Figure 6, the expression of GDH increased with time after infection. The levels of GDH were over 28% higher in infected cells than in mock-infected cells at 12 and 16 h p.i., respectively. Similarly, the expression of CAD increased substantially at 4 h p.i., and remained elevated throughout the course of infection. The level of CAD in infected cells increased by over 80% and 115% at 12 and 16 h p.i., respectively. In contrast, GLS expression was not altered significantly during infection. These findings suggest that GDH and CAD may be upregulated during viral infection.

**Figure 7:**
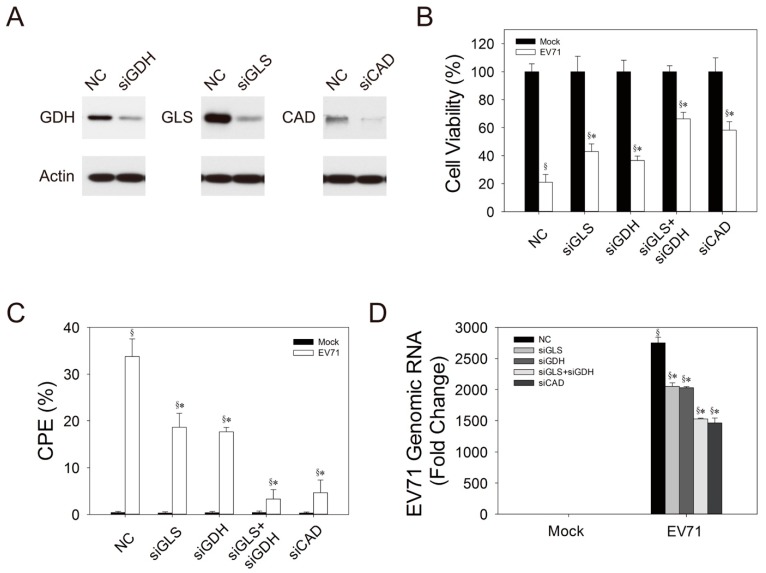
GLS, GDH, and CAD are essential to viral replication. (**A**) Vero cells were transfected with NC siRNA, siGDH, siGLS and siCAD, and 48 h after transfection, the cells were analyzed for expression of GDH, GLS and CAD by western blotting. An equal amount of the lysate of each transfectant was analyzed for expression of actin by western blotting. A representative result of three experiments is shown. (**B**–**D**) Vero cells were transfected with NC siRNA, siGDH, siGLS or siCAD for 48 h, and the transfected cells were mock- (solid bar) or infected (empty bar) with virus at MOI of 1.25. After 48 h, the infected cells were analyzed for cell viability (**B**) and the percentage of CPE (**C**). The cell viability data are expressed as percentage relative to those of mock-infected cells. Data are mean ± SD of six experiments. (**D**) Total RNA was extracted from mock- (Mock) or infected (*EV71*) cells for determination of viral genomic RNA. The result is expressed as fold change relative to that at 0 h p.i. Data are mean ± SD of six experiments. ^§^ *p* < 0.05, significant difference from mock-infected cells; * *p* < 0.05, significant difference from NC siRNA-transfected cells.

To test if these glutamine metabolism-related enzymes play important roles in viral replication, we transfected Vero cells with siRNA against GLS, GDH and CAD; infected them with EV71; and evaluated the effect of knockdown on the host cell viability and viral replication. As shown in Figure 7A, transfection of Vero cells with siRNAs effectively reduced the expression of GLS, GDH and CAD. Transfection with GLS siRNA (siGLS) or GDH siRNA (siGDH) alone caused an increase in the relative viability of the infected cells (Figure 7B; for the effect of siRNAs on the growth of transfected cells, please refer to Figure S3). Transfection with siGLS and siGDH in combination caused further increase in this parameter (Figure 7B). It was associated with reduction in CPE and viral replication. Transfection with GLS siRNA (siGLS) or GDH siRNA (siGDH) per se resulted in 45% or 48% decrease in the percentage of CPE in infected cells, and transfection with siGLS and siGDH in combination caused a 90% reduction in this parameter. The levels of viral genomic RNA were reduced by 25.5%, 26.3% and 44.5% in infected siGLS-, siGDH-, and the doubly transfected cells, respectively. These findings suggest that GLS and GDH most likely contribute to viral replication via the supply of αKG. Likewise, transfection with siCAD increased the relative viability of infected cells (Figure 7A). It was accompanied by an 86.3% decrease in the percentage of CPE (Figure 7B), and a 46.8% decrease in the level of genomic RNA (Figure 7C). These findings suggest that CAD may be essential to viral replication.

### 3.6. EV71 VP1 Protein Interacts with CAD

**Figure 8:**
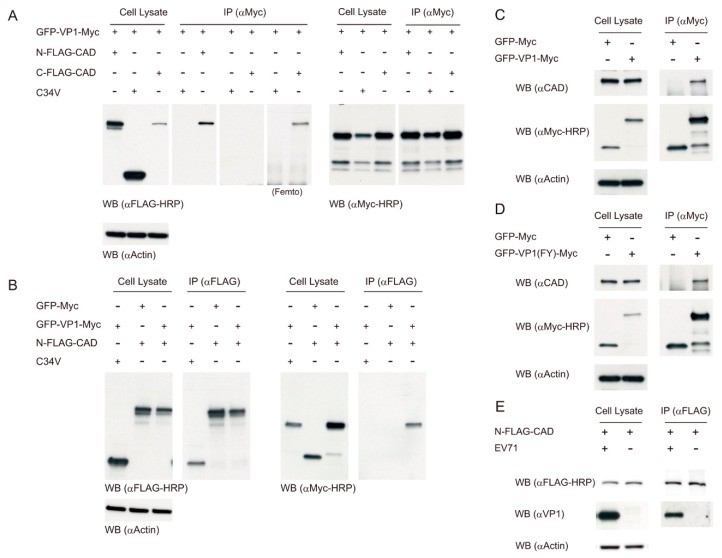
Interaction between VP1 and CAD. (**A**) Vero cells were transfected with the plasmid encoding GFP-VP1-Myc and that coding for N-FLAG-CAD, C-FLAG-CAD, or C34V. Forty-eight hours later, the cell lysate was prepared for immunoprecipitation with anti-c-Myc antibody (αMyc; c-Myc monoclonal antibody 9E10), and the subsequent western blotting with HRP-conjugated anti-FLAG antibody (αFLAG-HRP; MA1-91878) for detection of the co-immunoprecipitated N- or C-FLAG-CAD, or with HRP-conjugated anti-c-Myc antibody (αMyc-HRP; R951-25) for detection of GFP-VP1-Myc. (**B**) Reciprocal immunoprecipitation with anti-FLAG antibody (αFLAG; FLAG M2 monoclonal antibody) was followed by western blotting with αMyc-HRP for detection of the co-immunoprecipitated GFP-VP1-Myc, or with αFLAG for detection of N-FLAG-CAD. The cell lysate was immunoblotted with αFLAG-HRP, αMyc-HRP or anti-actin (αActin) antibodies. A representative result of three experiments is shown. (**C**,**D**) Vero cells were transfected with expression vector of GFP-VP1-Myc (**C**), GFP-VP1(FY)-Myc (**D**) or GFP-Myc (negative control) for 48 h, and the cell lysate was subjected to immunoprecipitation with αMyc and western blotting with anti-CAD antibody (αCAD) or with αMyc-HRP. The cell lysate was immunoblotted with αCAD, αMyc-HRP and αActin. A representative result of three experiments is shown. (**E**) Vero cells were transfected with the plasmid encoding N-FLAG-CAD for 48 h, and the transfected cells were infected with EV71 at MOI of 1.25 for 24 h. The cell lysate was subjected to immunoprecipitation with αFLAG and western blotting with anti-VP1 antibody (αVP1) and αFLAG. The cell lysate was immunoblotted with αVP1, αFLAG-HRP and αActin. A representative result of three experiments is shown.

To study the viral proteins that bind to host proteins to regulate metabolism, we employed a proteomic approach to screen for those cellular proteins interacting with viral proteins. We constructed a series of vectors encoding the c-Myc-tagged fusion proteins of green fluorescent protein (GFP) and EV71 proteins. The immunoprecipitation was performed with anti-c-Myc antibody (c-Myc monoclonal antibody (9E10)), and any co-immunoprecipitated proteins were subjected to proteomic analysis. One of these co-immunoprecipitated proteins was identified as CAD. To validate such interaction, we transfected Vero cells with expression vectors encoding GFP-VP1-Myc and FLAG-CAD (either N-FLAG-CAD or C-FLAG-CAD), and performed immunoprecipitation with c-Myc monoclonal antibody, or reciprocally with FLAG M2 antibody. The immunoprecipitated proteins were detected with anti-c-Myc and anti-FLAG antibodies. As shown in Figure 8, the expression level of C-FLAG-CAD in Vero cells was lower than that of N-FLAG-CAD. Immunoprecipitation with anti-c-Myc antibody revealed that GFP-VP1-Myc co-immunoprecipitated with N-FLAG-CAD and C-FLAG-CAD, but not with the control protein C34V. It appeared that the interaction of C-FLAG-CAD with GFP-VP1-Myc was weaker than that of N-FLAG-CAD (Figure 8A). Reciprocal immunoprecipitation with anti-FLAG antibody yielded similar results (Figure 8B). These findings suggest that VP1 and CAD may interact in EV71-infected cells.

We sought to examine whether VP1 interacts with endogenous CAD. Vero cells were transfected with expression vectors encoding GFP-VP1-Myc and GFP-Myc, and the cell lysate was immunoprecipitated with anti-c-Myc antibody. The immunoprecipitate was analyzed for the presence of endogenous CAD. As shown in Figure 8C, GFP-VP1-Myc was co-immunoprecipitated with endogenous CAD. Similar finding was obtained with GFP-VP1(FY)-Myc derived from VP1 cDNA of another EV71 strain (Fuyang, FY) (Figure 8D). Moreover, we transfected Vero cells with vectors to express N-FLAG-CAD, and infected these transfected cells with EV71. Cell lysate was prepared for immunoprecipitation with anti-FLAG antibody and subsequent western blotting with anti-VP1 antibody (αVP1). The Figure 8E shows that N-FLAG-CAD bound to the native VP1 protein of EV71. These findings substantiate the interaction between VP1 and CAD.

### 3.7. Exogenous VP1 Expression and Infection Increase the Flux of CAD-Catalyzed Reactions

**Figure 9:**
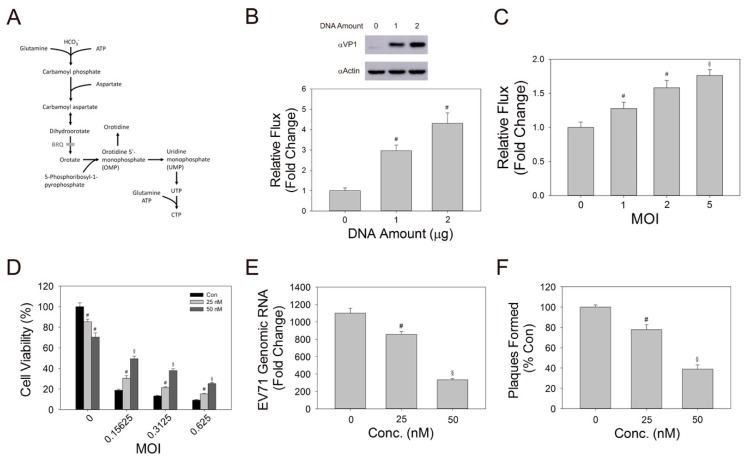
VP1 expression promotes pyrimidine biosynthesis that is essential to viral replication. (**A**) A simplified diagram shows the de novo pyrimidine biosynthesis pathway. It is noted that BRQ selectively inhibits DHODH. (**B**) (Upper panel) Vero cells were transfected with 0, 1, or 2 μg of VP1 expression vector (plus empty expression vector to maintain the total amount of transfected DNA at 2 μg). Twenty-four hours later, cells were harvested for western blotting with anti-VP1 antibody (αVP1) or anti-actin (αActin) antibodies. A representative result of three experiments is shown. (Lower panel) Vero cells were transfected in a similar manner. Twenty hours later, cells were labeled in NaH^13^CO_3_-containing medium and treated with BRQ as described in Materials and Methods. The cell extracts were subjected to mass spectrometric analysis. The abundance of [^13^C]-dihydroorotate (*m*/*z* 158.0283) was quantified from the corresponding extracted ion chromatogram and divided by the labeling time, and the flux is expressed as fold change relative to that of mock-transfected cells. Data are mean ± SD of six replicates. ^#^*p* < 0.05, significant difference from the mock-transfected cells. (**C**) Vero cells were infected with EV71 at MOIs of 1, 2 and 5, labeled in NaH^13^CO_3_-containing medium, and treated with BRQ, as described in Materials and Methods. The abundance of [^13^C]-dihydroorotate was quantified and divided by the labeling time, and the flux is expressed as fold change relative to that of mock-infected cells. Data are mean ± SD of six replicates. ^#^ *p* < 0.05, ^§^ *p* < 0.01, significant difference from the mock-infected cells. (**D**) Vero cells were mock- or infected with EV71 at the indicated MOIs for 1 h, and treated with 25 or 50 nM BRQ till 48 h p.i. The cell viability was assayed, as described above. The data are expressed as percentage relative to those of the untreated mock-infected cells, and presented as mean ± SD of six separate experiments. ^#^ *p* < 0.05, ^§^ *p* < 0.01, significant difference from the untreated cells (Con). (**E**) Vero cells were mock- or infected with EV71 at MOI of 0.3125 for 1 h, and treated with 25 or 50 nM BRQ till 30 h p.i. Total RNA was extracted for quantification of genomic RNA. The result is expressed as fold change relative to that at 0 h p. i. The data are presented as mean ± SD of six separate experiments. ^#^ *p* < 0.05, ^§^ *p* < 0.01, significant difference from the untreated cells. (**F**) Vero cells were infected with EV71 for 1 h, and were overlaid with 0.3% agarose in MEM/2% FCS, which was supplemented with 25 or 50 nM BRQ. The infected cells were processed for crystal violet staining as described in Materials and Methods. The number of plaques was counted, and is expressed as percentage relative to that formed in the absence of BRQ. Data are mean ± SD of six experiments. ^#^ *p* < 0.05, ^§^ *p* < 0.01, significant difference from that formed in the control plates.

CAD is a trifunctional protein with enzymatic activities necessary for the first three reactions of de novo pyrimidine biosynthesis (depicted in Figure 9A). The enzyme utilizes glutamine, bicarbonate, ATP and aspartate to form dihydroorotate, which is further acted upon by another enzyme DHODH to generate orotate. Normally, the steady-state level of dihydroorotate is very low. To measure the flux of CAD-catalyzed reactions, we adopted an inhibitor approach, and cultured cells in medium containing NaH^13^CO_3_ in the presence of BRQ, a potent and selective DHODH inhibitor. In this manner, the newly synthesized [^13^C]-dihydroorotate accumulated in cells, and the rate of its accumulation is indicative of the average flux of CAD-catalyzed reactions. To study if VP1 increases such flux, we transfected Vero cells with the expression vector of VP1, labeled the transfected cells with NaH^13^CO_3_, and subsequently treated with BRQ. The labeled cells were extracted for determination of [^13^C]-dihydroorotate. As shown in Figure 9B, exogenous expression of VP1 enhanced the CAD-catalyzed reactions. To study whether EV71 infection causes an increase in the flux of CAD-catalyzed reactions, we infected cells with EV71 at different MOIs, and incubated the infected cells with NaH^13^CO_3_ in the presence of BRQ. The labeled cells were extracted and analyzed accordingly. Infection increased the relative flux of CAD-catalyzed reactions in an MOI-dependent manner (Figure 9C). These results suggest that VP1 may interact with CAD to elevate the flux of de novo pyrimidine biosynthesis.

To validate that de novo pyrimidine biosynthesis is essential to viral replication, we infected cells with EV71 at different MOIs, treated them with BRQ and evaluated its effect on the viability of infected cells and the extent of viral replication. Despite the effect of BRQ on the viability of mock-infected cells, it protected cells from the virus-induced viability loss at all MOIs tested. The protective effect appeared to be dependent on BRQ concentration (Figure 9D). Consistent with such findings, BRQ treatment significantly reduced the level of EV71 genomic RNA (Figure 9E), and plaque formation (Figure 9F). These findings suggest that the de novo pyrimidine biosynthesis is essential to viral replication.

## 4. Discussion

Our present study demonstrates that viral infection induces metabolic reprogramming of host cells. A number of metabolic pathways, including glutathione metabolism, glycolysis and tricarboxylic acid cycle, and pools of several amino acids were altered in the infected cells. Of these pathways, the glutamine/glutamate catabolism is critical to viral replication. Depletion of glutamine in medium was inhibitory to viral replication. The glutamine-catabolizing enzymes GLS/GDH and CAD play important roles to meet the metabolic needs of viral replication, as a knockdown of these enzymes reduced CPE and viral genomic RNA synthesis. VP1 was found to interact with CAD and to regulate the flux of CAD-catalyzed reactions. The present findings suggest that metabolic reprogramming occurs in host cells in response to viral infection, and certain metabolic pathways, such as de novo pyrimidine synthesis, are upregulated by host-viral protein interaction.

Viruses rely on host metabolism to support their replication. It is reasoned that the host metabolism is reprogrammed by virus to its own benefit. Energy metabolism is upregulated. There is an increase in utilization of glycolysis, as evident by a rise in abundance of FBP, the product of rate-determining step of glycolysis (Figure 3). In addition to glycolysis, the intracellular glutamine level remained steady at low MOIs and only decreased slightly at an MOI of 1.25. In contrast, the glutamate level increased with MOI (Figure 2 and Figure 3). These findings imply that glutamine may be readily taken up from the medium, most likely via transporter solute carrier family 7 member 5 (Y+ system cationic amino acid transporter) known to be present in Vero cells [40]. Glutamine is converted to glutamate by GLS, and glutamate is subsequently oxidized to αKG by GDH. The maintenance of αKG and succinate pools in face of drastic depletion of citrate and oxaloacetate suggests that glutaminolysis is critical to a continual supply of αKG (Figure 3). Such notion is substantiated by the effect of silencing of GLS and GDH expression (Figure 7).

Additionally, DM-αKG treatment partially substituted for glutamine in supporting viral replication. In agreement with this, glutaminolysis is upregulated in adenovirus, human cytomegalovirus (HCMV)-, hepatitis C virus (HCV)-, and Kaposi’s sarcoma-associated herpesvirus (KSHV)-infected cells [41,42,43]. The changes in other TCA intermediates are also noteworthy. The levels of fumarate and malate were largely maintained at low MOIs, and decreased considerably at an MOI of 1.25. This implies that these metabolites may be consumed, especially at high MOI, to replenish oxaloacetate, which can be converted to aspartate via transamination. Additionally, the GDH reaction and a part of TCA cycle from αKG to oxaloacetate may be employed for energy production and provision of macromolecular precursors. Aspartate is required for de novo purine and pyrimidine biosynthesis. As it can be derived from oxaloacetate through transamination, depletion of the oxaloacetate pool implies its excessive conversion to aspartate and utilization for purine and pyrimidine biosynthesis. Moreover, the apparent paradoxical reduction in pyruvate level (in light of an increase in FBP level) suggests an increase in conversion of pyruvate to such metabolites as citrate or oxaloacetate. Owing to the presence of low level of oxaloacetate, the amount of pyruvate converted to citrate is most likely small compared to that diverted to anaplerotic reactions. Pyruvate can be converted to oxaloacetate through pyruvate carboxylase in an ATP-dependent manner [44], or through the combined actions of malic enzyme and malate dehydrogenase [45]. Moreover, the extent of reduction in the level of citrate was lower than that of oxaloacetate, suggesting that an alternative pathway may produce citrate. It is known that αKG can be converted to citrate through isocitrate dehydrogenase I (IDH1)-mediated reductive carboxylation [46]. Such reaction may occur in the enterovirus-infected cells to furnish citrate, in a manner similar to what has been observed in adenovirus-infected cells [47].

Enteroviral infection induces mitochondrial generation of reactive oxidative stress [23]. Consistent with such findings, the GSSG/GSH ratio increased in an MOI-dependent manner. Interestingly, the GSH level increased in the infected cells. One of its constituent amino acids, cysteine, increased in abundance at low MOI, but it decreased at high MOI. It is likely that cysteine accumulates intracellularly due to uptake of cysteine from medium, leading to an increase in its level at low MOI. However, its excessive utilization at high MOI outpaces the supply, and reduces the level of cysteine. The abundance of γ-glutamyl cysteine, an intermediate in GSH biosynthesis, changed with a similar trend. Glycine, which is not present in medium, decreased significantly with MOI and became depleted at an MOI of 1.25. These findings suggest that the infected cells may upregulate their biosynthesis of GSH, which is excessively oxidized to disulfide. 

There exist specific changes in amino acid pools in the EV71-infected cells. Several amino acids, including glutamine, glycine and aspartate, are involved in purine and pyrimidine biosynthesis. Glycine that is consumed in biosynthesis of purine and GSH decreased in abundance. The level of aspartate that is used for purine and pyrimidine biosynthesis decreased in a similar manner. In contrast, the intracellular level of glutamine whose concentration in medium is 2 mM was largely maintained in the infected cells. The glutamine level decreased modestly only in the cells infected at high MOI. As mentioned in the preceding section, glutamate may arise from GLS-catalyzed reaction. Other pathways, such as histidine catabolism, may also contribute to intracellular glutamate pool. Apart from the dehydrogenation reaction, glutamate may participate in transamination of oxaloacetate and pyruvate to aspartate and alanine, respectively. Consistent with our findings, changes in amino acid pools have been found in echovirus 30-infected cells [22]. Interestingly, glycine and asparagine in cerebrospinal fluid, when combined with phosphatidylcholine, can be used as biomarker classifier for enteroviral meningitis [48].

There are specific changes in γ-glutamyl dipeptides in the infected cells. The γ-Glu-Leu level was reduced in an MOI-dependent manner, whereas the γ-Glu-Ala level showed an opposite trend (Figure 3). The γ-glutamyl dipeptide can be generated through the activities of γ-glutamylcyateine synthetase (GCS) and γ-glutamyltranspeptidase 1 (GGT1). GCS catalyzes the rate-determining step of GSH biosynthesis. It has been shown that GCS can catalyze the conjugation of glutamate with amino acids other than cysteine and form γ-glutamyl dipeptides [49,50]. The presence of these dipeptides is generally considered to reflect an increase in GCS activity. It is apparent that no correlation exists between the levels of different γ-glutamyl dipeptides. Specific changes in their abundance cannot be attributed merely to the availability of particular amino acids. This implies that the selectivity of GCS may be somehow regulated to bring about specific changes in γ-glutamyl dipeptides. Additionally, GGT1 is expressed on the cell surface, and is involved in glutathione salvage pathway. GGT1 catalyzes the breakdown of GSH, GSSG and GSH-conjugates, which are exported extracellularly. The γ-glutamyl group is transferred to such acceptor molecules as amino acids. The γ-glutamyl dipeptides are taken up through specific transporters, and converted by γ-glutamyl cyclotransferase and 5-oxoprolinase to glutamate [39]. It is possible that the increase in γ-Glu-Ala may represent the preferential use of Ala for glutathione salvage. The biological roles of γ-glutamyl dipeptides are not clear. These compounds have been found to be food constituents that elicit taste and smell sensation. The γ-L-Glu-Val and γ-Glu-Leu are determinant of taste of bean [51]. The composition of γ-Glu-Gly, γ-Glu-Leu, γ-Glu-Met, γ-Glu-Phe, γ-Glu-Tyr, γ-Glu-His, γ-Glu-Gln, and γ-Glu-Glu affects the flavor of cheese [52,53]. Changes in γ-glutamyl dipeptides have been associated with chromophobe renal cell carcinoma [54], and with various liver diseases, such as drug-induced hepatic injury, HBV infection, HCV infection, cirrhosis, and hepatocellular carcinoma [55]. Recently, it has been shown that γ-Glu-Val exerts anti-inflammatory and anti-septic effect against bacterial infection [56]. The anti-inflammatory response may be mediated via activation of calcium-sensing receptor, promotion of β-arrestin-TAB1 interaction, and inhibition of TAK1–TAB1 complex formation [57]. It is plausible that the specific changes in glutamyl dipeptides may affect the antiviral response of host cells. 

In the present study, a host cell model in which virus replicates efficiently was employed. Vero cells are epithelial cells derived from African green monkey, and are able to support robust replication of EV71. For other cell types known to be infected by EV71 [30,58], studies are under way to study if similar metabolic changes occur in these cells. It is wondered if the metabolites found in Vero cells might differ from those of human cells. We have compared the metabolite profiles of a number of human and non-human primate cell lines (our unpublished data). There appears to be quantitative differences in abundance of metabolites but not qualitative differences in their chemical nature across the cell lines tested. 

Host cells obtain pyrimidine nucleotides via de novo biosynthetic and salvage pathways [59]. During infection, the viral pathogen takes over such pathways to produce copious amounts of nucleotides for its replication. De novo biosynthesis begins with CAD that utilizes glutamine, aspartate, bicarbonate, and ATP to form dihydroorotate. Dihydroorotate is converted by mitochondrial DHODH to orotate in a coenzyme Q-dependent manner [60]. The CAD enzyme catalyzes three initial steps of biosynthesis, and its carbamoyl phosphate synthetase II activity represents the rate determining step of de novo biosynthesis [59,61]. The increased CAD expression in EV71-infected cells may contribute to an increase in pyrimidine supply. CAD expression can be upregulated through c-Myc activation [62], and MAPK kinase activation [63]. c-Myc activation itself can be regulated by MAPK-mediated phosphorylation [64]. Intriguingly, EV71 infection results in MAPK phosphorylation [32,65,66]. It is likely that the MAPK signaling pathway may be involved in upregulation of expression and/or activation of CAD. Besides, we have shown that VP-1 directly interacts with CAD, and enhances the flux of CAD-catalyzed reaction. It is not unprecedented that viral protein can bind to CAD. Adenoviral preterminal protein has been found to complex with CAD [67]. The Ebola virus VP24 associates with CAD [68]. It is currently unknown whether VP1 directly modulates the CAD activity, or indirectly does so through recruitment of additional regulatory factors of CAD.

Apart from its role in necleocapsidation, viral capsid proteins have other functions in viral replication. Hepatitis C virus (HCV) core protein binds to RNA-dependent RNA polymerase to inhibit its RNA synthetic activity [69]. Brome mosaic virus capsid protein binds to 5’UTR of genomic RNAs to repress translation [70]. It is not as unexpected as it might appear at first sight that viral capsid protein modulates the activity of metabolic enzyme. Previous study has shown that expression of HCV core protein enhances the expression and activity of fatty acid synthase [71]. Our finding, showing that EV71 VP1 binds to CAD and alters its activity in the infected cells, is in line with such a notion, and is suggestive of an important role of VP1 in regulation of pyrimidine metabolism. Expression of VP1 may promote the pyrimidine de novo biosynthesis so as to maintain a continual supply of nucleotides for viral replication.

## Supplementary Materials

The following are available online at https://www.mdpi.com/2073-4409/9/2/473/s1, Figure S1: Effect of glutamine on growth of Vero cells, Figure S2: Effect of dimethyl-α-ketoglutarate (DM-αKG) on growth of Vero cells; Figure S3: Effect of siGDH, siGLS and siCAD on growth of Vero cells, Table S1: Metabolites differentially abundant in EV71-infected cells.
