# Supplementary material for: Metabolic Reprogramming of Host Cells in Response to Enteroviral Infection
Source: Cells. 2020 Feb 18;9(2):473. doi: 10.3390/cells9020473 (PMC7072837; doi:10.3390/cells9020473)
Supplement: Supplementary file 1 [file cells-09-00473-s001.zip › Supplementary_Files_R/Supplementary_Information.docx]

**Supplementary Information**

**Figure S1.** Effect of glutamine on growth of Vero cells. Vero cells were cultured in medium containing the indicated concentrations of glutamine (Gln) for 24 or 48 h, and the cell number was determined by neutral red assay. The data are expressed as fold change relative to those of untreated cells at 24 h, and presented as mean ± SD of six separate experiments. *p < 0.05, significant difference from 24 h at a specified concentration.





**Figure S2.** Effect of dimethyl-α-ketoglutarate (DM-αKG) on growth of Vero cells. Vero cells were cultured in medium containing the indicated concentrations of DM-αKG for 24 or 48 h, and the cell number was determined by neutral red assay. The data are expressed as fold change relative to those of untreated cells at 24 h, and presented as mean ± SD of six separate experiments. *p < 0.05, significant difference from 24 h at a specified concentration.





**Figure S3.** Effect of siGDH, siGLS and siCAD on growth of Vero cells. Vero cells were transfected with NC siRNA, siGDH, siGLS and siCAD, and 48 h after transfection, the cell number was determined by neutral red assay. The data are expressed as fold change relative to those of NC-transfected cells, and presented as mean ± SD of six separate experiments. *p < 0.05, significant difference from NC group.

**Supplementary Table Legend**

**Table S1.** Metabolites differentially abundant in EV71-infected cells. The metabolites that were differentially abundant in EV71 cells infected at different MOIs are tabulated. The metabolite identification (ID) numbers, names, the forms of adducts detected, mass-to-charge (m/z) ratios, retention times, intensities (at different MOIs), and HMDB ID of these metabolites are shown.
